# Accuracy of prenatal detection of facial clefts and relation between facial clefts, additional malformations and chromosomal abnormalities: a large referral-center cohort

**DOI:** 10.1007/s00404-023-07084-8

**Published:** 2023-06-16

**Authors:** Florence Vibert, Guel Schmidt, Kerstin Löffler, Adam Gasiorek-Wiens, Wolfgang Henrich, Stefan Verlohren

**Affiliations:** 1 Department of Obstetrics, Charité – Universitätsmedizin Berlin, Campus Charité-Mitte, Charitéplatz 1, 10117 Berlin, Germany; 2 Department of Oral and Maxillofacial Surgery, Charité Universitätsmedizin Berlin, Campus Virchow-Klinikum, Augustenburger Platz 1, 13353 Berlin, Germany

**Keywords:** Cleft lip, Cleft palate, Ultrasound, Prenatal diagnosis, Diagnostic accuracy, Chromosomal abnormalities, Additional malformations

## Abstract

**Purpose:**

Facial clefts belong to the most common congenital malformations and their prenatal diagnosis is a constant challenge. The aim of this study was to determine the accuracy of prenatal ultrasound in correctly classifying facial clefts. Furthermore, we aimed to specify the distribution of the type of clefts and underlying genetic conditions.

**Methods:**

All fetuses seen with suspected facial cleft in the Department of Obstetrics, Charité – Universitätsmedizin Berlin during a period of 23 years (1999–2022) were included in this retrospective study. Clefts were classified according to the classification of Nyberg. All additional prenatal findings were assessed and correlated with the outcome. The accuracy of prenatal diagnosis was assessed.

**Results:**

292 patients were included in the study. The most common type of clefts were unilateral cleft lip and palate (CL-P) (53.6%) and bilateral CL-P (30.6%), followed by CL (8.1%), CP (5.1%) and median CL-P (2.6%). The overall pre- and postnatal concordance rate corresponding to a correct prenatal diagnosis was high, 88.9%, ranging from 73.7% (CL) to 93.7% (unilateral CL-P). Most of the median clefts (95.2%) and CP (93.3%) were associated with other sonographic abnormalities, as well as 52.2% of bilateral CL-P. Chromosomal abnormalities, mostly trisomy 13 and trisomy 18, were observed in in the median CL-P (47.6%), bilateral CL-P (31.1%) and CP (26.7%) groups, in contrast to the CL (9.1%) and unilateral CL-P (12.9%) groups. It was exceptional to have a chromosomal abnormality without additional malformations (4.8%). The mortality rate including one late miscarriage, 5 IUFD’s, 74 TOPs and 6 palliative cares at birth was 29.8%, particularly high for median clefts (90.5%).

**Conclusion:**

Prenatal ultrasound exhibited a high accuracy to assess the type of facial clefts with an average rate of 88.9% (73.7%–93.7%) and a concordance rate of up to 93.7%, depending on the type of cleft. The search for additional malformations as well as clarifying underlying genetic conditions is essential. This allows for a targeted counseling of the parents and to best prepare for postnatal care, including surgery by the maxillofacial team.

## What does this study add to the clinical work

**Table:** 

We demonstrate, in a large cohort, the high accuracy of prenatal ultrasound in assessing the type of facial clefts (detection of correct type of cleft of 88.9 % (73.7 %–93.7 %)). An accurate diagnosis of the type of cleft allows physicians to inform and advise the parents in the best way possible, in particular on the scope of further genetic testing, but also to prepare the best postnatal care.	

## Introduction

Prenatal diagnosis of clefts is a challenge for the obstetrician to be able to inform and counsel the parents, but also to best prepare for postnatal care, which most often includes surgery. In Germany, three basic scans in first, second and third trimester are performed by primary care obstetricians. When a fetal abnormality is suspected but also when the primary care provider sees an indication for a more detailed scan, the woman is referred to a higher level prenatal diagnostic center in order to perform a very detailed ultrasound, and, when necessary, other diagnostic procedures.

Facial clefts, including cleft lip (CL), cleft lip with cleft palate (CL-P) and cleft palate (CP) are among the most common congenital malformations [1, 2]. The overall prevalence is 9.92 per 10.000 births. A higher prevalence is found in Japan (20.04 per 10.000), in Western Europe (12.10 per 10.000) and in the United States (10.20 per 10.000) as compared to Eastern and South-Mediterranean Europe [3]. The prevalence differs by sex, race and maternal age. Environmental and genetic risk factors are known to be involved in the development of facial clefts [4]. Facials clefts can be isolated or associated with additional malformations, especially when the palate is affected. Thus, many fetuses with an antenatal suspicion of clefts will not reach term due to associated lethal malformations. They will therefore be at risk of dying in utero or soon after birth, or undergo termination of pregnancy [5]. Non-isolated clefts are often part of a syndrome and/or are associated with a chromosomal abnormality [2].

The aim of this study was to determine, in a large cohort, the accuracy of prenatal ultrasound and the concordance between antenatal and postnatal diagnosis for the different types of facial clefts. Furthermore, we aimed to specify the distribution of the type of clefts and underlying genetic conditions.

## Methods

All fetuses seen with suspected facial cleft in the Department of Obstetrics, Charité – Universitätsmedizin, a tertiary referral center in Berlin, during a period of 23 years (1999–2022) were included in the retrospective study. The ultrasound was performed by a senior Maternal–Fetal Medicine Specialist. Both 2D and 3D images were analyzed. The presence of a cleft lip was suspected by the visualization of a uni- or bilateral defect on 2D frontal or axial slices, but also by 3D images, the integrity of the palate was assessed on 2D axial images by looking for the "equals sign", corresponding to an unremarkable uvula, whenever possible.

The suspected diagnoses were classified following the classification of Nyberg et al. Type 1 corresponded to isolated CL, which was defined by a cleft in the soft tissues of the upper lip. CL-P type 2 or 3 were diagnosed if both lip and palate were involved. The cleft could be unilateral (type 2), or bilateral (type 3). Type 4 was characterized by a midline CL-P, and type 5 by facial or atypical clefts associated with an amniotic band syndrome [6]. There is no Nyberg classification for isolated cleft palates, which are increasingly detectible in prenatal ultrasound. However, they are part of other classifications, such as the LAHSAL classification, an acronym for a sequence of descriptors “Lip, right—Alveolus, right—Hard palate—Soft palate—Alveolus, left—Lip, left”. [7]

The following variables were assessed based on an analysis of the ultrasound reports: maternal and gestational age at diagnosis, fetal gender, additional malformations including not only major, but also minor malformations as described in previous studies [8] and chromosomal or genetic abnormalities. Pierre Robin sequence was classified as associated CP. When a cleft was diagnosed, the parents were counselled on further genetic testing, and if chromosomal abnormalities were detected, a genetic consultation was recommended. The invasive diagnostic technique could be either chorionic villus biopsy or amniocentesis, during which a sample of placental tissue or amniotic fluid, respectively, was taken under ultrasound guidance. The result of the FISH test, as well as of the culture were recorded.

Postnatal data were obtained from hospitalization or operation reports of children managed by the Berlin Department of Cleft Lip and Palate at Charité hospital. Late-miscarriages, intrauterine fetal death (IUFD), terminations of pregnancy (TOP) and palliative cares were noted and, when available, autopsy records were reviewed. Pre- and postnatal data were compared in order to determine the accuracy of prenatal diagnosis for the different types of clefts. The study was approved by the Internal Review Board.

## Results

**Fig. 1:**
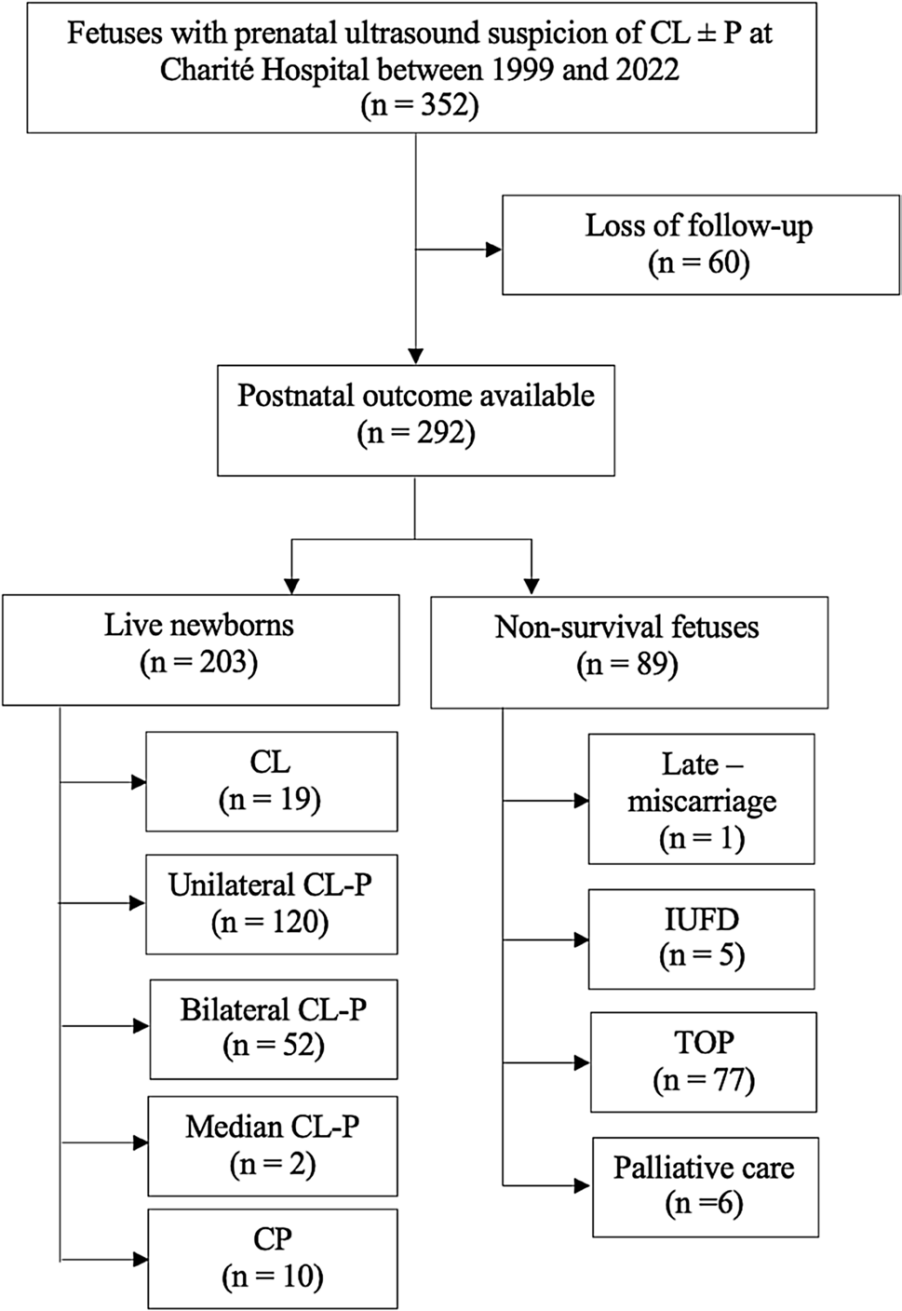
Flow-chart of the study. *CL* cleft lip; *CL*-P cleft lip palate; *CL ± P* = cleft lift with or without cleft palate; *CP* cleft palate; *IUFD* intrauterine fetal death; *TOP* termination of pregnancy

**Fig. 2:**
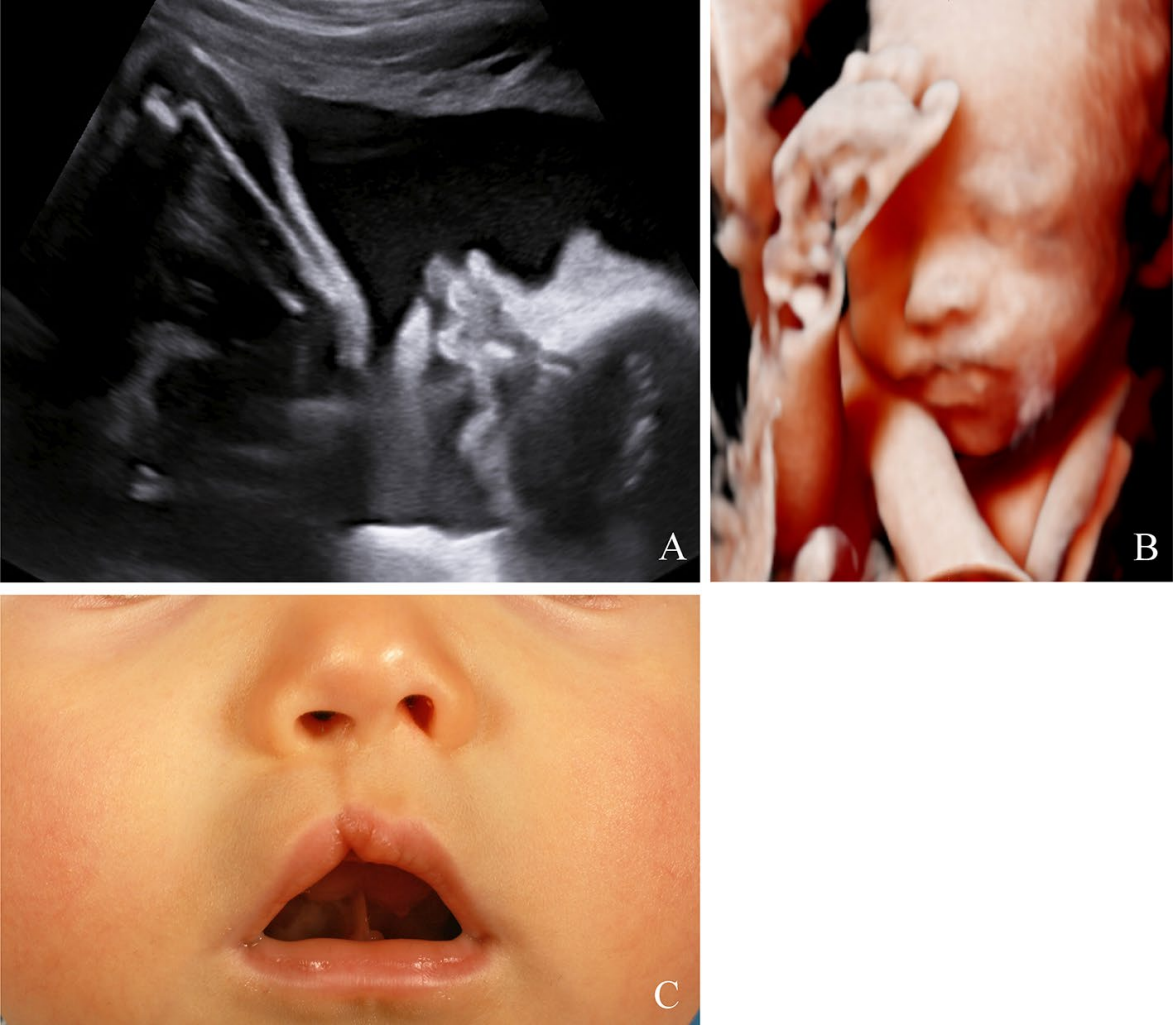
Unilateral cleft lip visualized on ultrasound during the prenatal period in 2D, in longitudinal section (**A**), and in 3D (**B**). Postnatal finding (**C**)

**Fig. 3:**
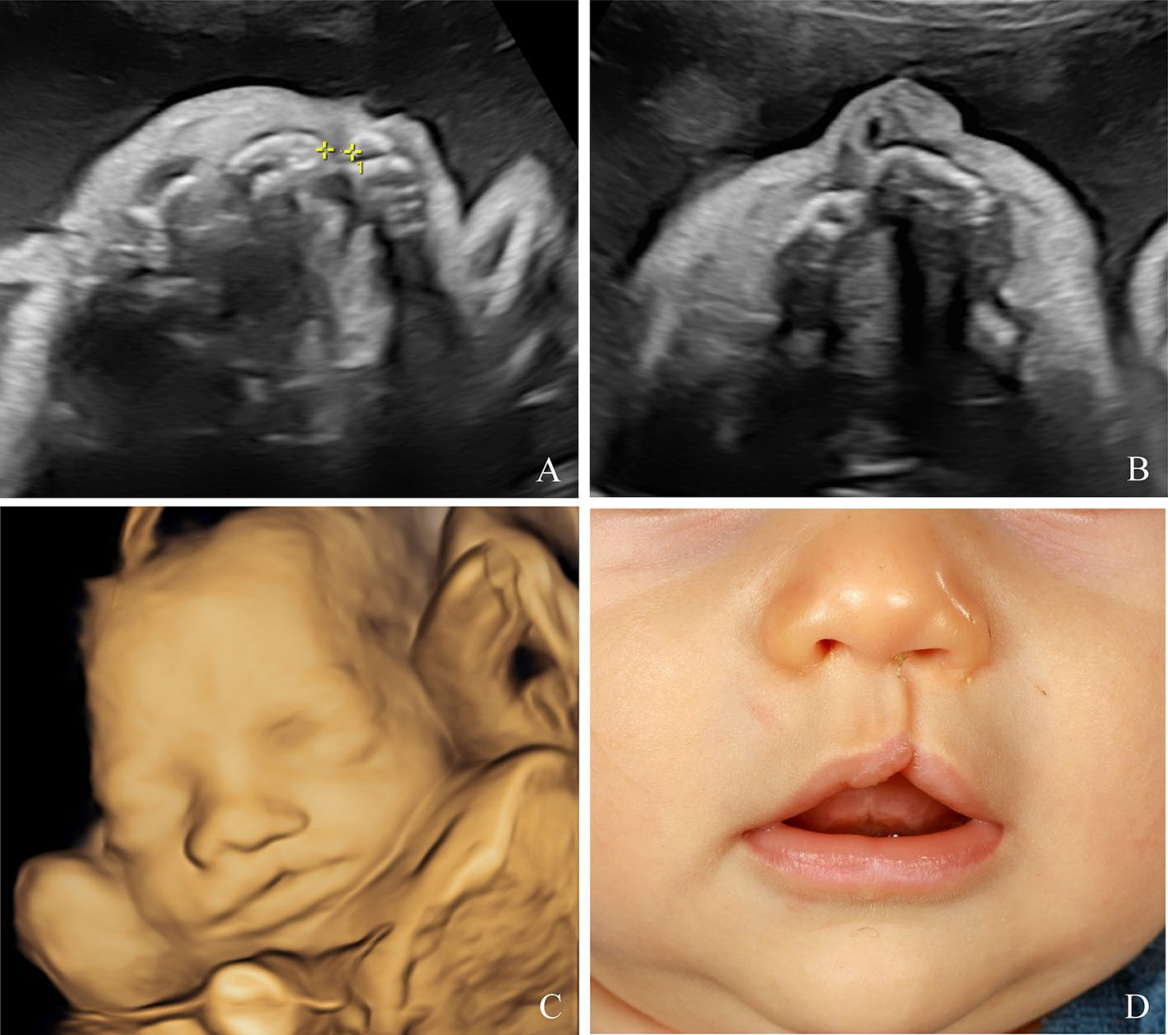
Unilateral cleft lip and palate, visualized on ultrasound during the prenatal period in 2D, in transverse sections (**A**, **B**), and in 3D (**C**). Postnatal finding (**D**)

**Fig. 4:**
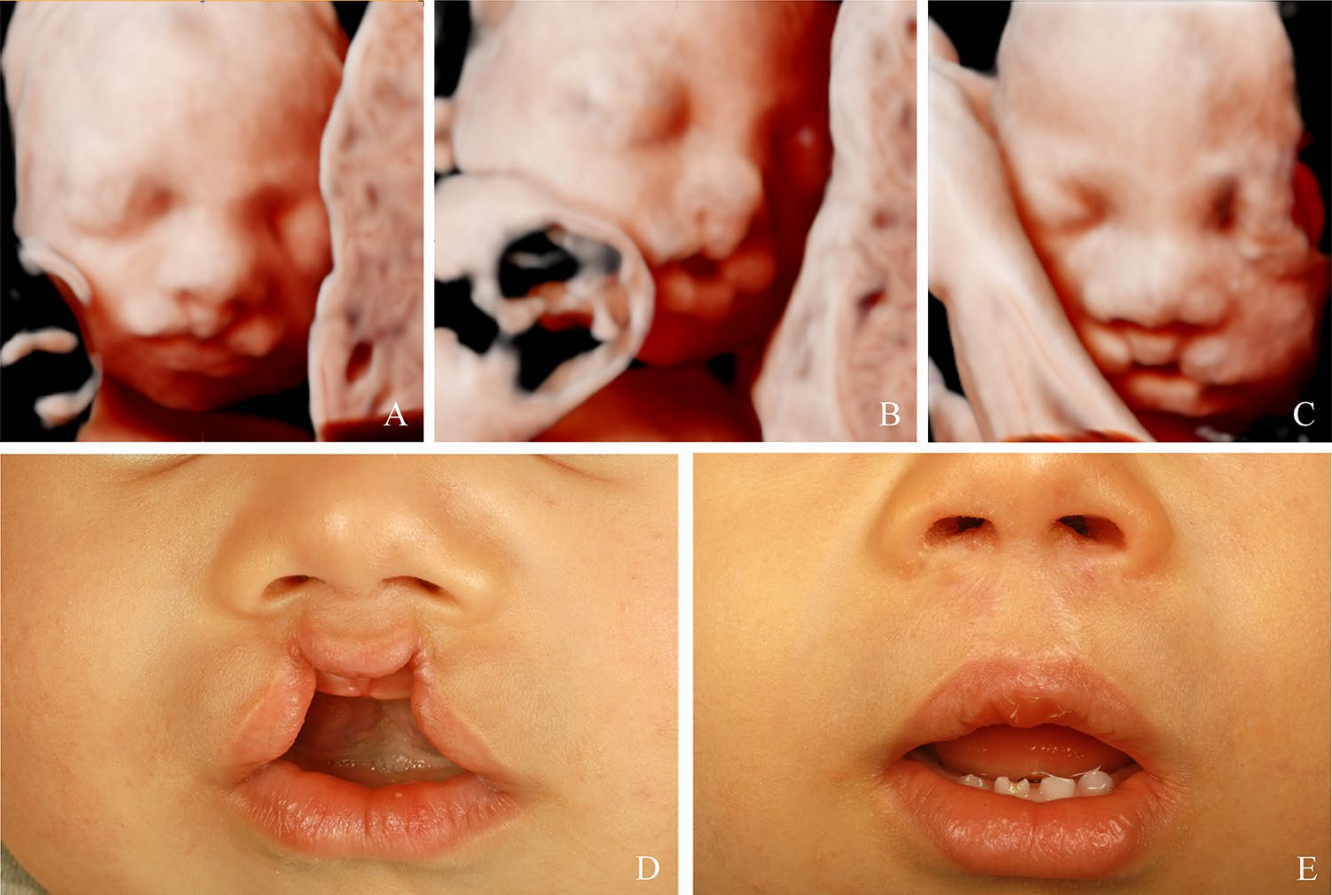
Bilateral cleft lip visualized on ultrasound during the prenatal period in 3D (**A**–**C**). Postnatal preoperative (**D**) and postoperative (**E**) result

**Fig. 5:**
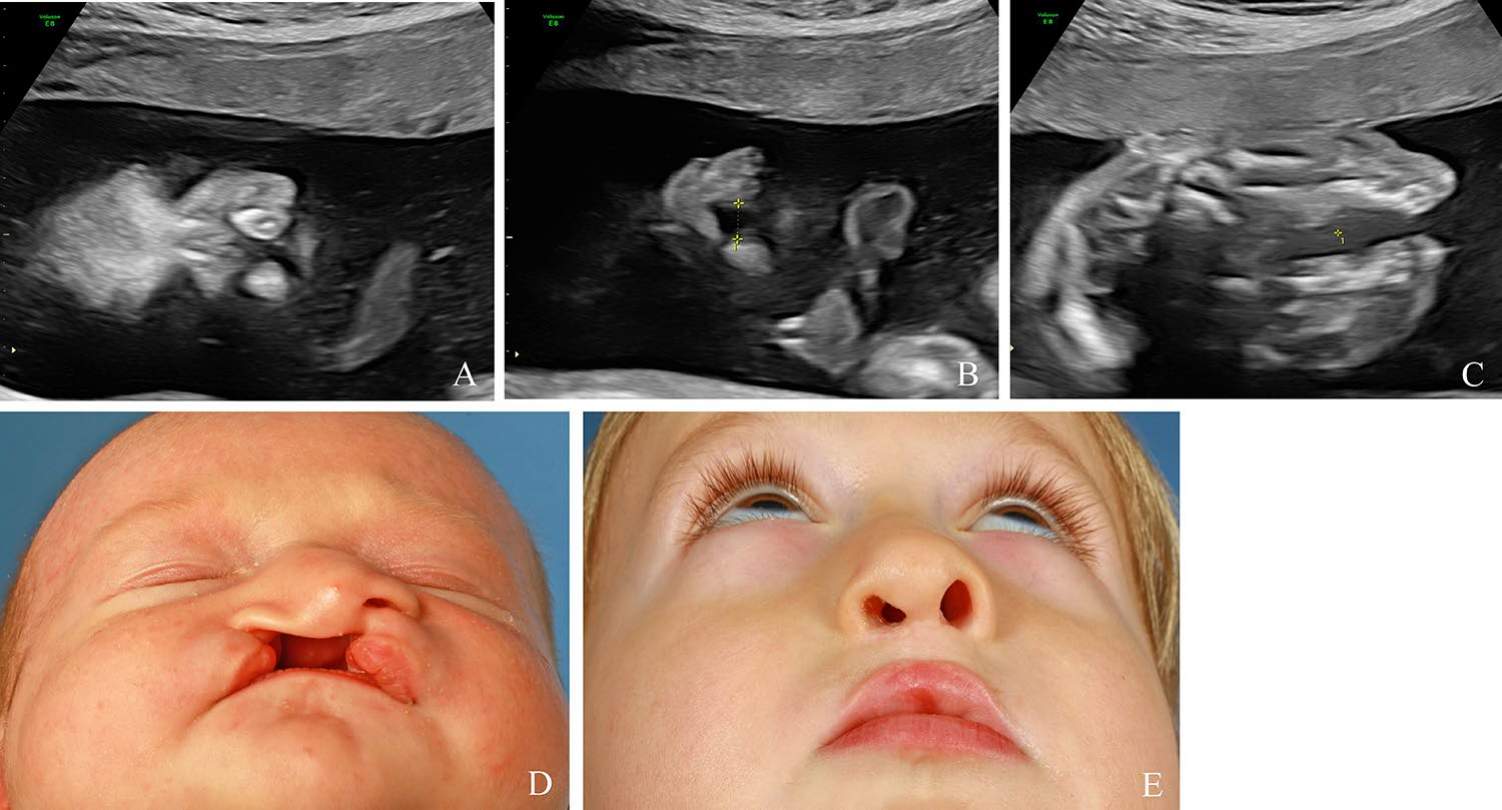
Midline hypoplasia and unilateral cleft lip and palate, visualized on ultrasound during the prenatal period in 2D, in frontal (**A**, **B**) and transverse (**C**) sections. Postnatal preoperative (**D**) and postoperative (**E**) result

**Fig. 6:**
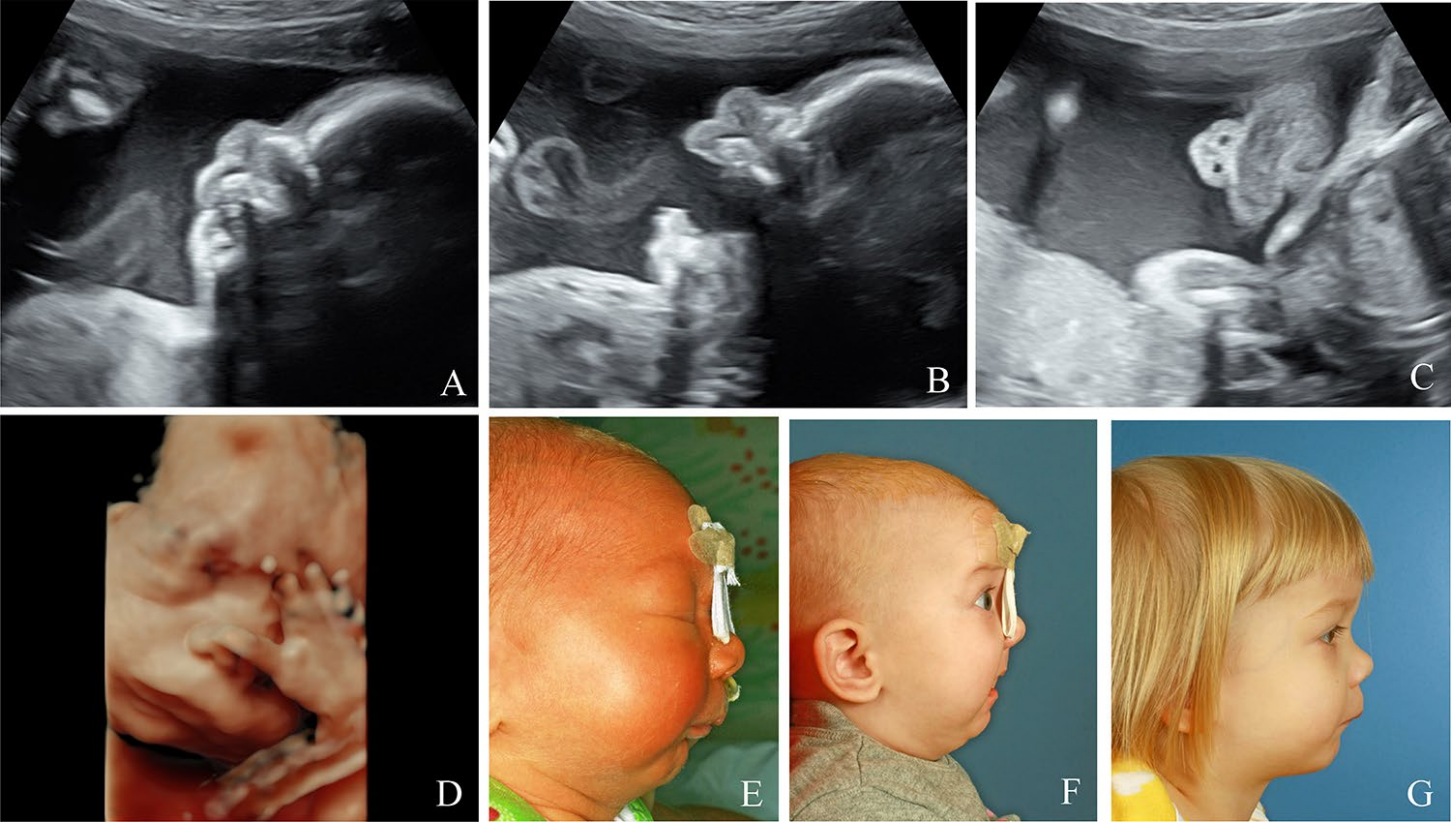
Pierre-Robin Sequence: Prenatal 2D (**A**–**C**) and 3D (**D**) images. Postnatal findings pre- and postoperative (**E**–**G**)

**Fig. 7:**
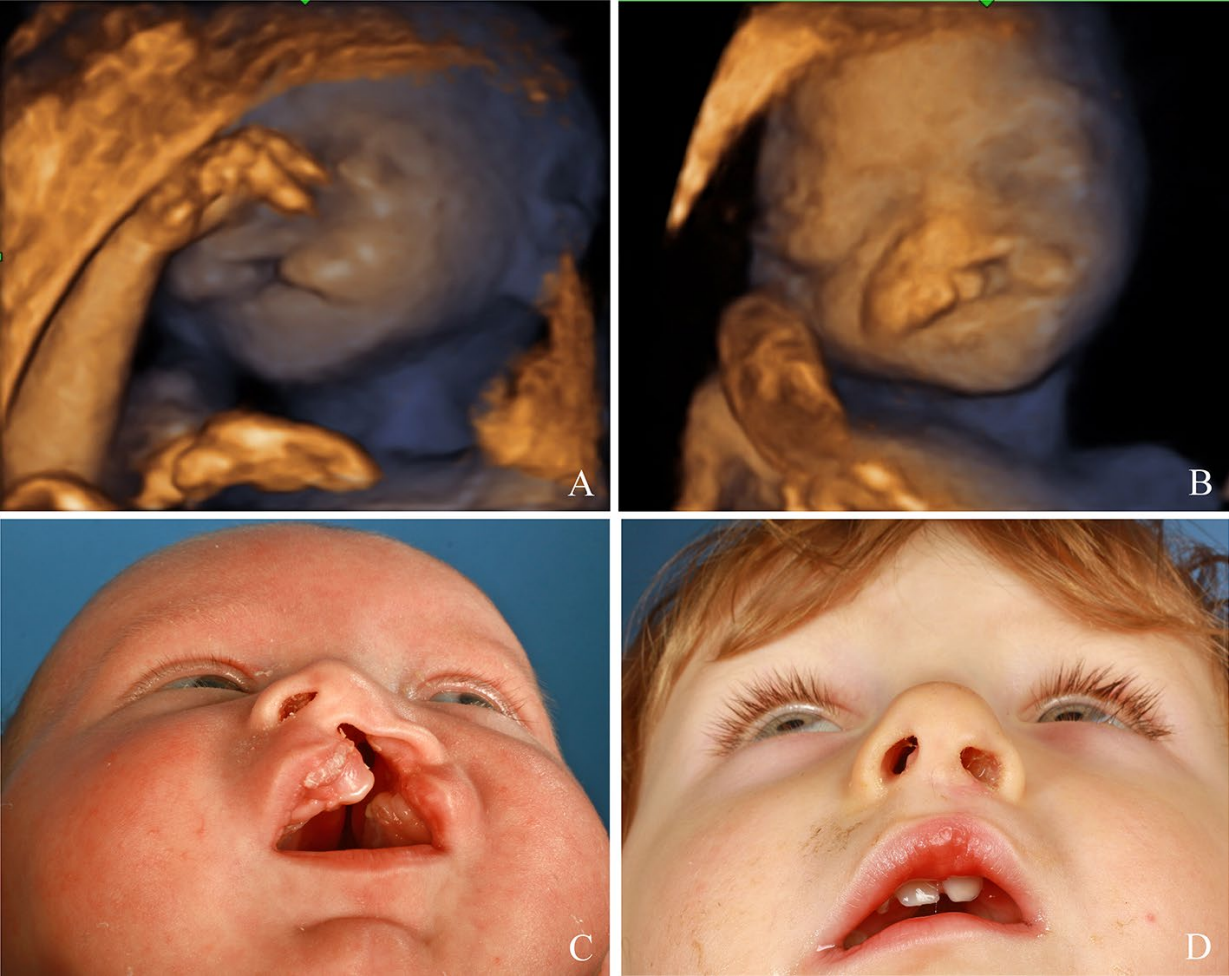
Cleft palate: Prenatal 2D (**A**, **B**) images. Postnatal findings pre- and postoperative (**C**, **D**)

A total of 352 patients with a prenatal suspicion of a facial cleft were included in the study. The postnatal outcome was available for 292 (83.0%) of them, corresponding to 203 live newborns and 89 non-viable fetuses (Fig. 1). 60 patients were loss to follow up. The baseline characteristics of the patients are presented in Table 1. At the time of the first appointment, the mean maternal age was 29.7 (± 5.8) years-old, with maternal age ranging from 15 to 44 years-old, and the mean gestational age was 24.8 (± 5.0) weeks of gestation (WG), with WG ranging from 13.2 to 39.1. The prevalence of clefts differed according to gender with 59.6% of cases involving a male fetus. The distribution of the clefts among the living newborns was the following: 19 CL (Figs. 2 and 3), 120 unilateral CL-P (Figs. 4, 5), 52 bilateral CL-P, 2 median cleft (Fig. 6) and 10 CP (Fig. 7). The other 89 pregnancies were marked by 1 late-miscarriage, 5 IUFD’s, 77 TOPs, and 6 palliative cares at birth (Fig. 1).

**Table 1:** Baseline characteristics of patients

Characteristics	Patients
Maternal age (years), mean ± SD (n)	29.7 ± 5.8 (292)
Gestational age (weeks of gestation), mean ± SD (n)	24.8 ± 5.0 (292)
Fetal sex (males), n (%)	124/208 (59.6)

### Accuracy of prenatal diagnosis

For 235 patients, including all living newborns and 32 non-viable fetuses, a postnatal record of the cleft type was available. Table 2 represents the number of clefts, the gestational age, and the pre- and postnatal concordance depending on the type of clefts among these patients. The most common type of clefts were unilateral CL-P (53.6%) and bilateral CL-P (30.6%), followed by CL (8.1%), CP (5.1%) and median CL-P (2.6%). The gestational age (GA) at diagnosis was in average 25.3 ± 4.9 WG. It was earlier for median CL-P (24.5 ± 3.8 WG) and later for CP (28.8 ± 4.8 WG). The overall pre- and postnatal concordance rate was 88.9%, ranging from 73.7% (CL) to 93.7% (unilateral CL-P). The accuracy was particularly high when both lip and palate were affected. In 6.0% the extension was underestimated: 7 CL appeared to be extended to the palate at birth, and 7 median clefts or unilateral CL-P appeared to be bilateral CL-P. In 2.6% the extension was overestimated: 5 CL-P were in reality CL and 1 CL-P was a CP. The false positive rate was equal to 1.3%. Indeed, prenatal diagnosis was incorrect in 3 cases; intact orofacial structures were found after birth while 1 unilateral CL-P and 2 CP were suspected on ultrasound. They were therefore excluded from further analyses. It should be noted that associated malformations were present in all 3 cases and a TOP had been performed. A subgroup analysis of the accuracy rate was performed. By dividing our study period into 4 comparable subperiods [1999–2004], [2005–2010], [2011–2016], [2017–2022], the following accuracy rates were found: 86.5%, 86.6%, 94.8% and 88.1% respectively.

**Table 2:** Pre- and postnatal concordance of facial clefts

Type of cleft	CL	Unilateral CL-P	Bilateral CL-P	Median CL-P	CP	Total
Number n (%)	19 (8.1)	126 (53.6)	72 (30.6)	6 (2.6)	12 (5.1)	235
GA at diagnosis (WG), mean ± SD	27.0 ± 4.9	25.0 ± 4.4	25.1 ± 5.4	24.5 ± 3.8	28.8 ± 4.8	25.3 ± 4.9
Pre- postnatal concordance (%)	73.7	93.7	87.5	83.3	75.0	88.9

*CL* cleft lip; *CL-P* cleft lip palate; *CP* cleft palate; *GA* gestational age; *WG* weeks of gestation

### Facial clefts and additional malformations

We analyzed the association between the type of cleft and additional malformations in 289 fetuses with known postnatal outcome, including 22 CL, 140 unilateral CL-P, 90 bilateral CL-P, 21 median clefts, 15 CP and 1 amniotic band syndrome (Table 3). The number of associated malformations was particularly high in the median cleft group, reaching 95.2%, followed by the CP group (93.3%) in which nine Pierre Robin sequences were noted. The rate of additional malformations was lower considering the unilateral CL-P (32.1%) and the CL (22.7%) groups. In the bilateral CL-P group, 52.2% of patients had non-isolated clefts. The most common anomalies associated with clefts were cardiac (n = 82), cerebral (n = 68), extremity (n = 59), facial (n = 41) and ocular (n = 35) anomalies. In 12 cases (4.2%), no associated anomaly was suspected during the antenatal period and the primary diagnosis was made after birth.

**Table 3:** Additional malformations, chromosomal abnormalities and mortality rate depending on the type of clefts

Type of cleft	CL	Unilateral CL-P	Bilateral CL-P	Median CL-P	CP	Amniotic band syndrome	Total
Additional malformations n (%)	5/22 (22.7)	45/140 (32.1)	47/90 (52.2)	20/21 (95.2)	14/15 (93.3)	1/1 (100)	119/289 (41.2)
Chromosomal abnormalities n (%)	2/22 (9.1)	18/140 (12.9)	28/90 (31.1)	10/21 (47.6)	4/15 (26.7)	–	62/289 (21.5)
Mortality rate n (%)	3/22 (13.6)	20/140 (14.3)	38/90 (42.2)	19/21 (90.5)	5/15 (33.3)	1/1 (100)	86/289 (29.8)

*CL* cleft lip; *CL-P* cleft lip palate; *CP* cleft palate

### Facial clefts and chromosomal abnormalities

For 168 fetuses, a final genetic diagnosis was available. A total of 62 fetuses (21.5%) had a chromosomal abnormality (Tables 3, 4). This rate was higher in the median cleft group (47.6%) compared to the CL (9.1%) and the unilateral CL-P (12.9%) groups. The percentage was 31.1% in the bilateral CL-P and 26.7% in the CP groups. In most of the cases the chromosomal abnormality was a trisomy 13 (31/62, 50.0%), followed by trisomy 18 (7/62, 11.3%; Table 4). Two Cri-du-Chat-Syndrome characterized by a 5p deletion were also found in the uni- and bilateral CL-P groups. The other genetic diagnoses included trisomy 21 (n = 1), trisomy 14 (n = 1), one TP63-mutation associated with ectrodactyly, ectodermal dysplasia, cleft lip/palate syndrome 3 (EEC3), sex chromosome anomalies such as Turner (n = 1), and Klinefelter syndromes (n = 1), microdeletions such as 22q11 in DiGeorge syndrome (n = 1). Genetic testing was performed antenatally in most cases. Most genetic abnormalities were detected with karyotyping. Only in a minority of the more recent cases, molecular genetic approaches were applied leading to additional sub-microscopic findings, such as in the case of the TP63-mutation. In only 5 cases, genetic testing was made postnatally with one case of trisomy 13, one microdeletion 1p36 syndrome, one Goldenhar syndrome, one Van der Woude syndrome and one Fryns syndrome. In only three of the cases with genetic findings, the cleft was isolated. This was the case for one unilateral CL-P, in a Bartter syndrome context, and for two bilateral CL-P, one in a Van der Woude syndrome context and the other one with a derivative chromosome 4.

**Table 4:** Type of clefts and chromosomal abnormalities

Karyotype	CL	Unilateral CL-P	Bilateral CL-P	Median CL-P	CP	Total
Done n(%)	11/22 (50.0)	73/140 (52.1)	64/90 (71.1)	14/21 (66.7)	6/15 (40.0)	168/288 (58.3)
T13 (n)	1	4	17	8	1	31
T18 (n)	1	2	1	1	2	7
5p deletion (n)	0	1	1	0	0	2
Others (n)	0	11	9	1	1	22
Anomalies among the patients that had karyotyping (%)	18.2	24.7	43.8	71.4	66.7	36.9

*CL* cleft lip; *CL-P* cleft lip palate; *CP* cleft palate

### Outcome

The overall mortality rate, including 1 late miscarriage, 5 IUFD’s, 74 TOPs and 6 palliative cares at birth, was 29.8%. Among the 74 TOPs, 71 fetuses presented associated malformations and 39 had a chromosomal abnormality. In the 3 cases of isolated cleft, a chromosomal anomaly was found in 2 fetuses. The result of the genetic analysis was not available for the last fetus. The type of suspected cleft was in 2 cases a CL, in 17 cases a unilateral CL-P, in 33 cases a bilateral CL-P, in 18 cases a median cleft, and in 4 cases a CP. The mortality rate was greater than 90% for median clefts (90.5%), including 18 TOP between 13.3 and 36 WG and 1 palliative care management. It was less than 15% for CL (13.6%). The mortality rates for the other types of clefts were as follows: 42.2% for bilateral CL-P, 33.3% for CP and 14.3% for unilateral CL-P.

## Discussion

In a large, single-center cohort, we demonstrated the distribution of cleft lip and palate types, and the high accuracy of ultrasound in detecting prenatal cleft lip with or without cleft palate with accuracy rates ranging from 73.7 to 93.7%. Accuracy was particularly high when both, lip and palate were affected. Most of the median cleft (95.2%) and CP (93.3%) were associated with further fetal malformations. A chromosomal abnormality was mainly observed in median CL-P (47.6%), bilateral CL-P (31.1%) and CP (26.7%) groups, in contrast to the CL (9.1%) and unilateral CL-P (12.9%) groups. The two most common chromosomal defects were trisomy 13 and 18. Only 4.8% of the fetuses with a chromosomal abnormality had no additional malformations. The overall mortality rate was 29.8%, particularly high in the case of median clefts (90.5%).

Prenatal diagnosis of fetal facial clefts remains a challenge though improving over time [9], in some regions due to the implementation of national screening programs [1, 10]. The classical distribution of cleft types in the literature is 25% CL, 50% CL-P and 25% CP [9] which is in contrast to our study with 8.1%, 86.8% and 5.1% respectively. This discrepancy may be explained by the timing of stating the final diagnosis: inclusion of prenatally suspected clefts and all postnatally diagnosed clefts versus inclusion of only prenatally suspected clefts followed by adjudication after birth. Indeed, the prenatal detection rate of cleft lip and palate varies between 9 and 100% [11], and also depends on the type of cleft. For example, the ultrasound diagnosis of cleft palate is complex and therefore the rate of cleft palate will be falsely underestimated in prenatally diagnosed cleft cohorts. In addition, only cases for which the diagnosis of the type of cleft was certain, i.e. for which we had a postnatal diagnosis, were kept for the calculation of these data in order to obtain robust accuracy rates, the main objective of our study. Thus, all living fetuses were retained but only 32 non-viable fetuses out of 89 which may have affected the distribution among the different cleft types.

A major challenge is the prenatal detection of isolated CP. In our cohort, 12 CP (5.1% of all clefts) were diagnosed before birth. In the literature, the prenatal detection of CP was variable ranging from 0 to 89%. Offerdal et al. and Gillham et al. did not find any CP during the prenatal period in their prospective and retrospective cohort respectively. The systematic review of Maarse et al. showed a prenatal detection rate from 0 to 22% in low-risk population studies using 2D ultrasound, and from 0 to 89% in high-risk women using 3D ultrasound [2, 9, 12]. In this context, new ultrasound signs have been described to improve their detection, such as the “equal sign” [13].

We have shown a high overall accuracy rate in determining the type of clefts. In order to have robust data, cases without a definite postnatal diagnosis were excluded, thus keeping all newborns alive but only 32 of the 89 nonviable fetuses, which may have affected to some extent the distribution between cleft types. The systematic review published by Maarse and al. in 2010 found variable detection rates with 2D ultrasound in low-risk patients ranging from 9 to 100% for CL with or without CP, 0 to 22% for CP, and 0 to 73% for all types of clefts. The detection rates with 3D ultrasound among high-risk women were higher: 100% for CL, 86 to 90% for CLP and 0 to 89 for CP [11]. A retrospective cohort showed an overall accuracy rate of 76.9%. The extension of the defect was more often underestimated (19.4%) than overestimated (3.7%), without error in distinguishing between uni- and bilateral clefts [14]. We were able to show a better pre- and postnatal concordance, and increasing with time, which are potentially explained by a constant improvement in the performance of ultrasound devices allowing increasingly accurate examinations, by better training and awareness of physicians in prenatal cleft screening, and by the introduction of new ultrasound evaluation techniques. Ji et al. demonstrated in 2021 the impact of 3D-ultrasounds in improving the accuracy of CL-P diagnosis [15]. Regarding the diagnosis of CP, Faure et al. found a high concordance rate with the use of a new ultrasound semiology including the use of a strict axial transverse ultrasound view (0.88, 95% CI 0.79–0.97) [16], while Lai and al. shown the value of 2D and/or 3D-ultrasound with a high pooled sensitivity (0.87, 95% CI 71%–95%) and specificity (0.98, 95% CI 90%–100%) [17]. The performance of fetal MRI also seems promising with a great pooled sensitivity (0.97, 95% CI 0.93–0.99) and specificity (0.94, 95% CI 0.89–0.97), which is, however, not relevant in a screening context [18].

Accurate prenatal diagnosis is paramount because of the vast psychological consequences for the parents following of the diagnosis of a fetal anomaly [19]. In addition, and in the context of clefts, an accurate prediction of its type helps to tailor counselling of the parents for the postnatal management by the interdisciplinary team and especially the pediatric maxillofacial surgery team. This varies greatly depending on whether only the lip and palate are affected or, for example in cases of Pierre Robin Sequence, a life threatening postnatal upper airway obstruction is expected. Most of all, and apart from the local defect, the prognosis of the newborn is dependent on the type of cleft. Harville et al. showed that in case of CL-P, associated anomalies were more common than in case of CL, but also that the newborns were more often premature with a low birth weight (p = 0.001). Moreover, they had a higher infant mortality rate (25/1.000 for CL-P and 15/1.000 for CL) although the difference was not statistically significant [20].

Regarding additional malformations, CL were more frequently isolated, in contrast to bilateral CL-P, but especially CP and median CL-P. This is in concordance to the published literature [12]. In a systematic review considering both, pre- and postnatal studies, Maarse et al. [2] found a low prevalence of associated anomalies for CL (0–20% in prenatal studies, 7.6–41.4% in postnatal studies) and a high prevalence for CL-P (39.1–66.0% in prenatal studies, 21.1–61.2% in postnatal studies). However, CP was the most frequently associated with other malformations (22.2–78.3% in postnatal studies). This rate is potentially dependent on who is performing the study. For example, members of maxillofacial surgery team will omit non-viable or aborted fetuses, which are more likely to have other anomalies. In addition, some authors exclude in their definition minor anomalies [21] or consider the Pierre Robin sequence differently, isolated, in a separate category or associated with CP [2]. We decided to include all malformations because some minor ones, such as nevi and undescended testes, can also be part of disorders like for example chromosomal aneuploidies, Noonan syndrome and Charge syndrome [21].

In concordance with the literature, chromosomal abnormalities were detected in 95.2% of the cases associated with additional malformations. A systematic review found in cases of isolated clefts a postnatal chromosomal defect rate of 1.8% for CL, 1.0% for CL-P and 1.6% for CP. Therefore, Maarse et al. recommend to adapt the prenatal advice, regarding the prognosis and the risk of chromosomal anomaly, on the type of clefts but especially on the presence or not of associated anomalies. According to them, an invasive genetic diagnosis should be considered in case of associated anomalies irrespective of cleft category. In case of isolated cleft, conventional karyotyping it not recommended for CL, but an array CGH to detect the 22q11.1 deletion should be considered [2, 22].

Our study has many strengths, particularly the large number of patients included and the possibility to verify the outcome as most fetuses received surgery in the Department of Cleft Lip and Palate, the biggest referral center for the management of newborns with facial clefts in Germany.

On the other hand, the study has limitations. This is a retrospective cohort, and 60 patients were lost to follow-up who may have been patients initially referred to the Charité hospital for a specialist opinion, but who delivered in their home region. In the case of a non-surviving fetus, the fetopathological examination was not always desired by the mother.

In conclusion, we have highlighted the high accuracy of prenatal ultrasound to assess the type of facial clefts. This allows the obstetrician to provide quality counseling to the parents and prepare for postnatal (emergency if needed) care, by the maxillofacial team. The search for additional malformations is an essential parameter in the decision to perform an invasive diagnosis since most chromosomal anomalies are associated with additional malformations. Further studies, especially prospective ones and follow-up studies are needed to complete these results.

## Data Availability

The data that support the findings of this study are available from the corresponding author, [SV], upon reasonable request.
